# Evaluation of CD160 and CD200 Expression as Differentiating Markers between Chronic Lymphocytic Leukemia and Other Mature B-Cell Neoplasms

**Published:** 2020-01-01

**Authors:** Wafaa Ahmed El- Neanaey, Rania Shafik Swelem, Omar Mohamed Ghallab, Sara Mohamed Abu-Shelou

**Affiliations:** 1 Department of Clinical and Chemical Pathology, Faculty of Medicine, University of Alexandria, Alexandria, Egypt; 2 Department of Internal Medicine (Hematology), Faculty of Medicine, University of Alexandria, Alexandria, Egypt

**Keywords:** CLL, Mature B-cell neoplasms, CD200, CD160, Flow cytometry

## Abstract

**Background: **The present work aimed to investigate the expression of CD160/ CD200 in CLL and other mature B-cell neoplasms (MBN) and their use as an additional diagnostic tool for differentiating CLL from other MBN.

**Materials and Methods: **Using flow cytometry, we detected the expression of CD160 &CD200 on B-cells from 30 CLL patients, 30 other MBN patients in addition to 20 controls. CDs160/200 measurements were determined as a percentage expression (≥20% was considered positive) and as a ratio of the mean ﬂuorescence intensities (MFIR) of leukemic cells/controls and were considered positive when the ratios were ≥2 and 20, respectively.

**Results: **90% and 100% of the CLL group expressed CDs160/200 in comparison to 60% and 63.3% of other MBN (p=0.007, p<0.001), respectively. By MFIR, 96.7% and 50% of our CLL group expressed CDs160/200 in comparison to 76.7% and 30% of other MBN, respectively. CDs160/ 200 were not expressed on the controls. Positive co-expression of CD160 and CD200 was found in 90% of the CLL cases, 60% of HCL patients and only in 40% of B-NHL. However, double negative expression of both markers was found only in 24% of the B-NHL patients.

**Conclusion** **: **CD160 with CD200 can be used as additional diagnostic markers to the available routine panel to differentiate between B-CLL and other non-specified B-NHL patients.

## Introduction

 B-cell chronic lymphoproliferative disorders (B-CLPD) include a heterogeneous group of disease entities arising from clonal proliferation of mature B-lymphocytes^1^. B-CLPD is now most often diagnosed by flow cytometric immunophenotyping that identifies a clonal light-chain restricted population expressing B-cell markers in the blood or BM ^2^. However, a final diagnosis cannot be done in all patients with B-CLPD by these methods ^3^.

Clinical features of CLL at presentation are heterogeneous, and a deﬁnitive diagnosis is based on the combination of peripheral blood B-cell lymphocytosis (≥5×10^9^/L), characteristic morphology, and immunophenotype. Matutes ﬂow cytometry score is particularly useful for differentiating between CLL and other MBN^4^^,^^5^ CLL classically has score of 4 or 5. However, some CLL cases may have an atypical immunophenotype and/or morphological features resulting in diagnostic confusion ^6^.

CD160 is an Ig- like activating natural killer (NK) cell receptor^7^^,^^8^.The CD160 gene is on chromosome 1q42.3^9^, expressed on most circulating NK cells and on a subset of circulating cytotoxic T cells, but not on B cells ^7^^,^^8^. Binding of CD160 to both classical and non-classical major histocompatibility complex class I enhances NK and CD8^+^cytotoxic-T lymphocytes functions^10^^-^^13^, as well as cytokine production, including IFN-ᵞ TNF-á, and IL6^11^^,^^12^. Recent work has demonstrated CD160 expression in malignant B cells ^14^.

CD200, is a type I glycoprotein that is expressed on thymocytes, activated T cells, B cells, dendritic cells, endothelial cells, and neurons but not on NK cells^15^^,^^16^. The CD200 gene is mapped to chromosome 3q13.2^17^^,^^18^. CD200 interacts with the CD200 receptor, which is confined to antigen presenting cells of myeloid origin and a subset of T cells^15^, resulting in immunosuppressive functions^19^.

CD200 expression was reported in CLL versus negative expression in MCL. The expression of CD200 was also reported in HCL, multiple myeloma, lymphoblastic lymphoma/leukemia, lymphoplasmacytic lymphoma, acute myeloid leukemias, and other non-hematologic malignancies^20^.

Recently, it has been reported that CDs160/200 were expressed in most cases of CLL^21^^,^^22^. In the absence of histological and/or cytogenetics/molecular additional explorations, the addition of CDs160/200 expression detection in atypical B-CLPD proliferative syndromes of uncertain diagnosis could help to reach a deﬁnitive conclusion and better orientate patients toward the most appropriate therapy.

**Aim of the work: **Our work aimed to investigate the expression of CDs160/200 in CLL and other MBN patients and their use as an additional diagnostic tool for differentiating CLL from other MBN.

## MATERIALS AND METHODS

 Our study was carried out on 60 newly diagnosed adult patients with CLPDs; according to the immunophenotypic analysis, patients had further diagnosis as 30 patients with CLL, 25 patients with B-NHL (4 mantle cell lymphoma (MCL) and 2 follicular lymphoma (FL) cases were included in this group) and 5 patients with HCL, who were recruited from the hematology unit in Main Alexandria University hospital during the period from November 2016 to October 2017. The diagnosis of CLL was based on the WHO 2016 diagnostic criteria according to standard criteria of microscopic cell morphology and ﬂow cytometry analysis. MCL diagnosis was confirmed by cyclin D1 by flow cytometry and by detecting t(11;14) by fluorescence in situ hybridization (FISH) . FL diagnosis was confirmed by CD10 expression by flow cytometry and by detecting t(14;18) by FISH. HCL diagnosis was confirmed according to morphological, clinical and speciﬁc ﬂow cytometric panel for HCL (CD103, CD11c, CD25). Approval of the Ethics Committee was obtained and a written informed consent was taken from all our participants.

All cases included in the study were subjected to full history taking ,thorough clinical examination and laboratory investigations including complete blood count in K2 EDTA tubes analyzed by automated cell counter ADVIA 2120 hematology system (Siemens Healthcare Diagnostics, serial number 10285573,Eschborn, Germany) and immunophenotyping for diagnosis using chronic flow cytometry panel adopted in our department including (CD5 FITC, clone UCHT2, cat no 555352, CD19PE, clone HIB19, cat no 555413,CD22 FITC clone HIB22, cat no 555424, FMC7 FITC, clone FMC7, cat no 332786, Kappa FITC, clone G20-193, cat no 555791, Lambda PE, clone JDC-12, cat no 555797,CD10PE, clone HI10a, cat no 555375 from BD Biosciences, USA&CD23 FITC-conjugated antibody, clone 9p25, cat no PN A07409; Beckman, USA) done for patients only using Becton Dickinson, FACS4-colors Calibur flow cytometer equipped with BD CellQuest Pro software (BD biosciences, San Jose, CA, USA).

In addition Human CD160 APC-conjugated Antibody, clone 688327 and Human CD200 percp-conjugated Antibody, clone 325516 from R&D systems- Biotechne, USA were added to our panel and analyzed for both patients and controls. 

Peripheral blood cells were processed for investigation of CD160 and CD200 expression using a combination of CD19, CD160, CD200 and CD5. 100μls of EDTA anti-coagulated PB were added to a falcon tube, and then, 10 μls of CD160/CD200/CD19/CD5 were added, mixed well with a vortex mixer and incubated for 10 minutes in the dark at room temperature. After incubation, the cells were washed twice with PBS, and centrifuged at 2000 rpm for 2 minutes each at room temperature, then two mls lysing solution were added to each sample, mixed gently and incubated for 10 minutes at room temperature in the dark, then the tubes were centrifuged for two minutes at 2000 rpm and the supernatant was discarded leaving approximately 50 µls of fluid, the cells were then washed twice with PBS. The supernatant was gently aspirated and discarded; leaving approximately 50µLs of fluid, the pellet was resuspended in 0.3 ml PBS for flow cytometric analysis. A negative control tube was submitted to all of the previous steps except the staining step. This was done to compensate for non-specific background of auto-fluorescence and to distinguish between fluorescent positive and fluorescent negative cell populations.

Data were acquired on BD FACSCalibur instrument. A four-color flow cytometer using CellQuest Pro software, 1x10^4^ events were acquired.

On interpretation of the CD160/ CD200 tube, a gate was set around the CD19 positive cells against side scatter and then we detected the percent of cells positive for CD5/19/160 and CD5/19/200 in both percentage and MFI if the cases were positive for CD5, and if the cases were negative for CD5; CD19/160 and CD19/200 percent and MFI were detected (supplementary Figure.).

Fluorescence intensity was measured using a logarithmic scale with signal intensity ranging from 10^0^ to 10^4^ arbitrary units. A ratio of MFI of cells incubated with anti CDs160/200 mAbs against the negative control was established. The positivity threshold of these ratios for B-cell was set at 2 and 20, respectively. Alternately, positivities for CDs160/200 were defined as at least 20% positive cells above the negative background fluorescence. 

Consistency in fluorescence was verified over the course of the study using Calibrite* beads (cat no: 340486; Becton Dickinson and company BD Biosciences San Jose, USA) gated according to the procedure recommended by the manufacturer.

Bone marrow aspiration or trephine biopsy was performed in patients, if necessary.

**Statistical analysis**

Data were fed into the computer and analyzed using IBMSPSS software package version 20.0***. *(**Armonk, NY: IBM Corp**)**. Significance of the obtained results was judged at the 5% level. 

Receiver operating characteristic (ROC) curves analysis was used to determine the predictive value of CD160/CD200.

## Results

Demographic data, Rai clinical staging system, clinical and laboratory data are presented in (Table 1). 

**Supplementary Fig:**
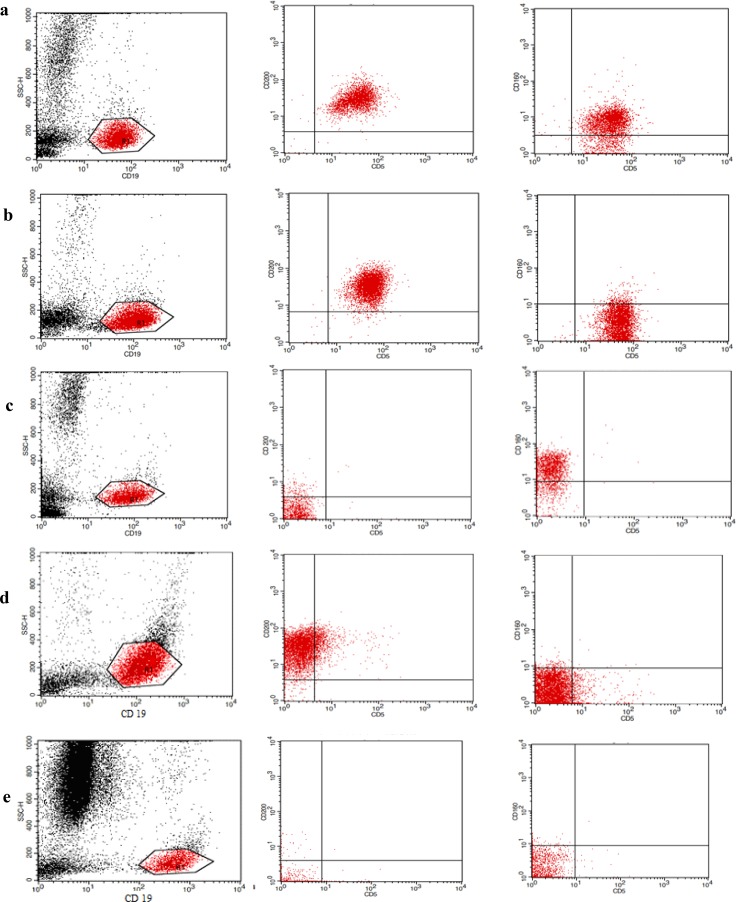
Flow cytometry dot-plots for CD160/200 expression in: (a) A CLL case showing double positivity for CD160/200. (b)A CLL case showing positivity for CD200 and CD160 negativity. (c) A B-NHL case showing positivity for CD160 and negativity for CD200. (d) A HCL case showing positivity for CD200and CD160 negativity. (e) A Control showing double negativity for CD200 and CD160.

**Table 1:** Demographic, laboratory and clinical data of the studied subjects

	**CLL** **(n = 30)**		**MBN** **(n = 30)**		**Control** **(n = 20)**		**p**
	**No.**	**%**	**No.**	**%**	**No.**	**%**	
Age (years)							
Min. – Max.	42.0 – 88.0		35.0 – 78.0		44.0 – 65.0		0.091
Mean ± SD.	59.93 ± 9.34		59.33 ± 9.92		54.4 ± 7.63		
Median	58.0		57.50		55.0		
Gender							
Male	16	53.3	17	56.7	14	70.0	0.482
Female	14	46.7	13	43.3	6	30.0	
Lymphadenopathy	24	80.0	18	60.0			0.091
Hepatosplenomegaly	18	60.0	21	70.0			0.417
B-symptoms	10	33.3	24	80.0			<0.001*
Hb (g/dl)							
Min. – Max.	4.40 – 14.0		3.50 – 13.0		11.50 –15.40		<0.001*
Mean ± SD.	11.35 ± 2.25		9.89^a^± 2.23		12.75^ab^± 1.07		
Median	11.75		10.0		12.55		
Platelets (10^3^/ul)							
Min. – Max.	28.0 – 397.0		8.0 – 424.0		200.0 – 450.0		<0.001*
Mean ± SD.	180.3 ± 86.90		102.6^a^± 78.72		275.4^ab^± 71.48		
Median	177.5		97.50		258.0		
WBCs (10^3^/ul)							
Min. – Max.	9.55 – 209.7		1.63 – 99.90		7.20 – 10.40		<0.001*
Mean ± SD.	42.36 ± 47.45		24.39^a^± 23.35		8.84^ab^± 1.06		
Median	25.75		15.94		8.95		
Lymphocytes (10^3^/ul)							
Min. – Max.	7.32 - 198.4		1.47 - 96.90		2.0 - 3.80		
Mean ± SD.	35.57 ± 45.85		19.73^a^± 21.98		2.94^ab^± 0.52		<0.001*
Median	19.67		12.14		2.90		
CD22							
Negative/ weak positive	27	29.0	7	23.3			<0.001*
Strong positive	3	10.0	23	76.7			
CD23							
Negative	1	3.3	23	76.7			<0.001*
Positive	29	96.7	7	23.3			
CD19							
Negative	0	0.0	0	0.0			**-**
Positive	30	100.0	30	100.0			
FMC7							
Negative	23	76.7	2	6.7			<0.001*
Positive	7	23.3	28	93.3			
Kappa							
Negative	13	43.3	14	46.7			<0.001*
Weak positive	15	50.0	2	6.7			
Strong positive	2	6.7	14	46.7			
Lambda							
Negative	17	56.7	16	53.3			0.018*
Weak positive	9	30.0	2	6.7			
Strong positive	4	13.3	12	40.0			
CD5							
Negative	0	0.0	21	70.0			<0.001*
Positive	30	100.0	9	30.0			
CD10							
Negative	30	100	28	93.3			0.492
Positive	0	0.0	2	6.7			
Matutes score							
0	0	0.0	10	33.3			<0.001*
1	0	0.0	13	43.3			
2	0	0.0	2	6.7			
3	6	20.0	5	16.7			
4	5	16.7	0	0.0			
5	19	63.3	0	0.0			
Rai staging							
Low	12	40.0					
Intermediate	12	40.0					**-**
High	6	20.0					

^a^: Significant with CLL ^b^: Significant with MBN

Quantitative data was expressed in mean ± SD, median (Min. - Max.) and compared using**F **ANOVA test or Kruskal Walli test**.** Qualitative data were described using number and percentage and was compared using Chi square or Monte Carlo test

* : Statistically significant at p ≤ 0.05CLL: chronic lymphocytic leukemia; MBN: mature B-cell neoplasms; Hb: hemoglobin

All healthy subjects had negative CDs160/CD200 expression levels on normal circulating B-lymphocytes with CD160 MFIR close to 1 and CD200 MFIR ranged from 6.28 to 11.06. The control group had statistical significant difference with all groups of patients as shown in (Table 2).

**Table 2:** Expression of CD160 and CD200 on B lymphocytes of healthy subjects and on leukemic cell clone of the patients

	**CLL** **(n = 30)**	**MBN (n = 30)**		**Control** **(n = 20)**	**p**
		**BNHL** **(n = 25)**	**HCL** **(n =5)**		
CD160 %					
Negative (<20)	3 (10%)	10 (40%)	2 (40%)	20 (100%)	<0.001*
Positive (≥20)	27 (90%)	15 (60%)	3 (60%)	0 (0%)	
Min. – Max.	0.0 – 99.12	0.0 – 96.67	0.0 – 95.95	0.0 – 1.74	
Mean ± SD.	70.14^b^± 30.12	42.42^ab^ ± 38	45.1^b^ ± 45.6	0.35 ± 0.54	
Median	83.62	50.57	43.5	0.09	
CD160 MFIR					
Negative (<2)	1 (3.3%)	5 (20%)	2 (40%)	20 (100%)	<0.001*
Positive (≥2)	29 (96.7%)	20 (80%)	3 (60%)	0 (0%)	
Min. – Max.	0.0 – 24.16	0.0 – 27.8	0.0 – 11.4	0.0 – 0.75	
Mean ± SD.	10.69^b^± 6.28	8.15^ab^ ± 7.34	5.21^b^ ± 5.57	0.29 ± 0.28	
Median	8.60	5.68	3.94	0.26	
CD200 %					
Negative (<20)	0 (0%)	11 (44%)	0 (0%)	20 (100%)	<0.001*
Positive (≥20)	30 (100%)	14 (56%)	5 (100%)	0 (0%)	
Min. – Max.	64.64 - 99.82	0.0 – 99.50	52.13 – 95.2	2.98 – 18.0	
Mean ± SD.	96.44^b^±6.44	44.8^ab^± 43.5	82.9^b^ ± 17.6	10.2± 4.62	
Median	98.45	33.4	90.08	4.61	
CD200 MFIR					
Negative (<20)	15 (50%)	19 (76%)	2 (40%)	20 (100%)	<0.001*
Positive (≥20)	15 (50%)	6 (24%)	3 (60%)	0 (0%)	
Min. – Max.	7.45 - 44.52	0.0 – 66.6	11.8 – 36.02	6.28- 11.06	
Mean ± SD.	20.85^b^±8.46	12.6^ab^ ± 14.4	20.3^b^ ± 9.77	8.40 ± 1.56	
Median	19.67	8.75	20.3	8.18	
CD160+ve%,CD160MFIR+ve	27^ b^ (90.0%)	15^ab^ (60.0%)	3^b^ (60.0%)	0(0.0%)	<0.001*
CD160+ve%, CD160MFIR-ve	0(0.0%)	0(0.0%)	0(0.0%)	0(0.0%)	–
CD160-ve%, CD160MFIR+ve	2(6.7%)	5(20.0%)	0(0.0%)	0(0.0%)	0.131
CD160-ve%, CD160MFIR-ve	1^ b^ (3.3%)	5 ^b^ (20.0%)	2^ab^(40.0%)	20(100.0%)	<0.001*
CD200+ve%, CD200MFIR+ve	15^b^(50.0%)	1^a^(4.0%)	3^b^(60.0%)	0(0.0%)	<0.001*
CD200+ve%, CD200MFIR-ve	15^b^(50.0%)	13^b^(52.0%)	2^b^(40.0%)	0 (0%)	<0.001*
CD200-ve%, CD200MFIR+ve	0(0.0%)	5^a^(20.0%)	0(0.0%)	0(0.0%)	0.011*
CD200-ve%, CD200MFIR-ve	0^b^(0.0%)	6^ab^(24.0%)	0^b^(0.0%)	20(100.0%)	<0.001*

^a^: Significant with CLL

^b^: Significant with Control

Quantitative data was expressed in mean ± SD, median (Min. - Max.) and compared using**F **ANOVA test or Kruskal Wallis test. Qualitative data were described using number and percentage and was compared using Chi square or Monte Carlo test

* : Statistically significant at p ≤ 0.05 MFIR: mean fluorescence intensity ratio;CLL:chronic lymphocytic leukemia ; MBN: mature B-cell neoplasms ;B-NHL :B-non hodgkin lymphoma ; HCL: hairy cell leukemia

CD160% expression was positive in 90% of CLL patients versus 60% of B-NHL and 60% of HCL patients, thus showing a statistical significance in B-NHL patients (p=0.009) and no statistical significant difference was found with HCL (p=0.139) (Table 2, Fig.1A).

As regards the CD160 MFIR, a statistical significance was found between CLL group and B-NHL group (p=0.048). No statistically significant difference was detected in HCL group (p=0.057) (Table 2, Fig.1A).

CD200 % expression was positive in 100% of CLL patients versus 56% of B-NHL and 100% of HCL patients, thus showing statistical significance in B-NHL patients (p<0.001) and no statistically significant difference was found in HCL (Table 2, Fig.1B).

As regards the CD200 MFIR, a statistical significance was found between CLL group and B-NHL group (p=0.048) and no statistically significant difference was detected in HCL group (p=1.000) (Table 2, Fig.1B).

**Figure1A:**
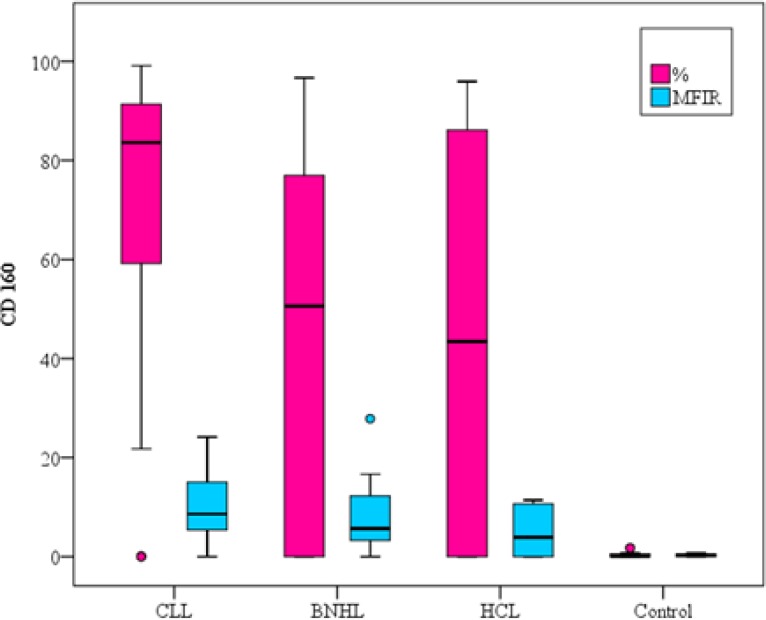
CD160 expression on different B-cell neoplasms and the control group

**Figure1B:**
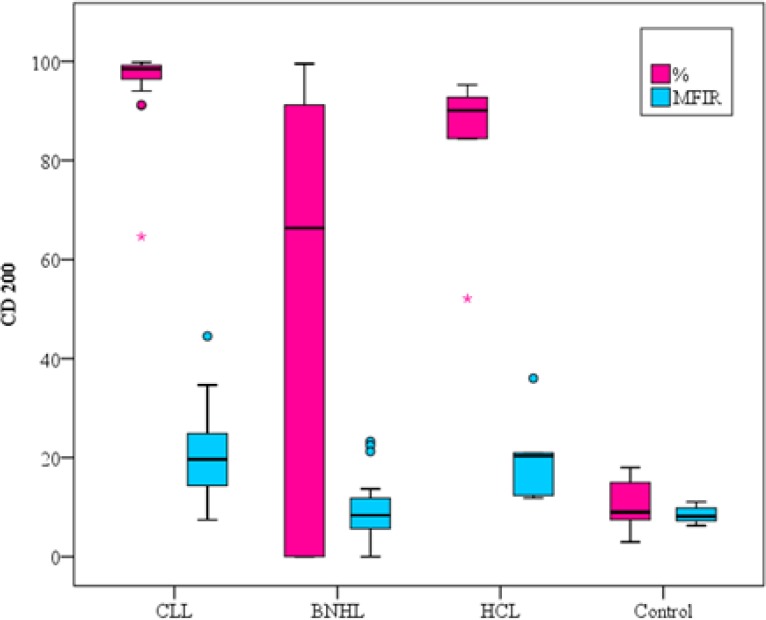
CD200 expression on different B-cell neoplasms and the control group

On classifying our patients, according to the co-expression of CD160/CD200, we relied only on the percentage positivity rather than the MFI ratio depending on the results revealed by our ROC curves as regards the sensitivity, specificity and AUC which were much more superior in the percentage rather than MFI ratio (sensitivity= 86.67%, 70%, specificity=80%, 63.33%, AUC=0.940, 0.724 respectively) (Fig. 2A, Fig. 2B).

**Figure 2A:**
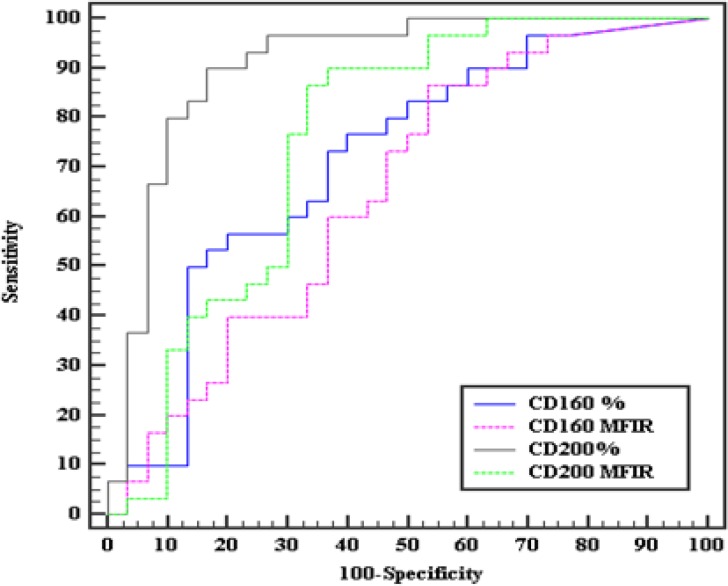
ROC curves of CD160 and CD200 for diagnosing CLL from the other MBN

**Figure 2B:**
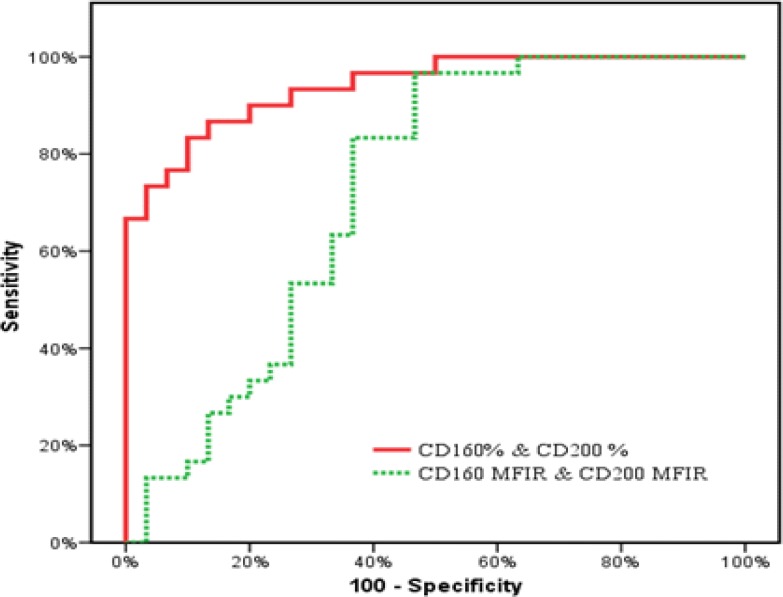
ROC curves for combined expression of CD160 & CD200 to diagnose CLL cases from the MBN.

Regarding co-expression of CDs160/200, it was double positive in 90% of CLL patients compared to 40% and 60% of B-NHL and HCL patients, respectively. While double negative expression of both markers was found only in 24% of B-NHL patients. Moreover, no double negative was seen in CLL and HCL patients (Fig.3A, Figure.3B).

**Figure 3A:**
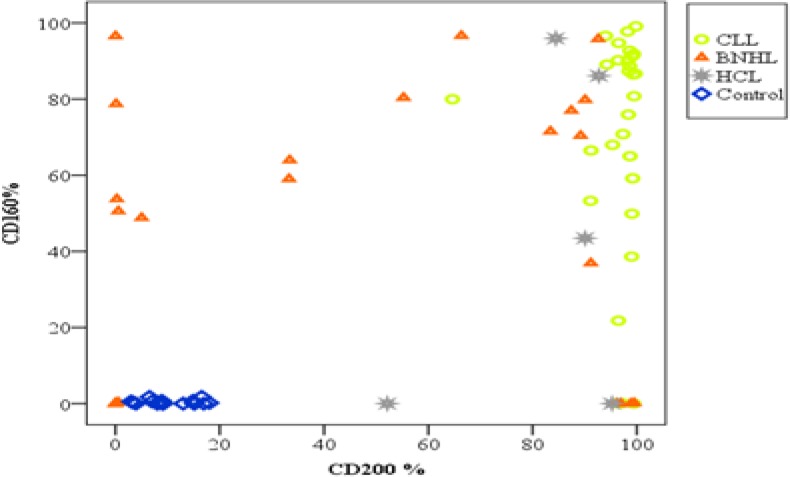
Distribution of patients (CLL and other MBN) and control according to CD 160 and CD200 percentage expression.

**Figure 3B:**
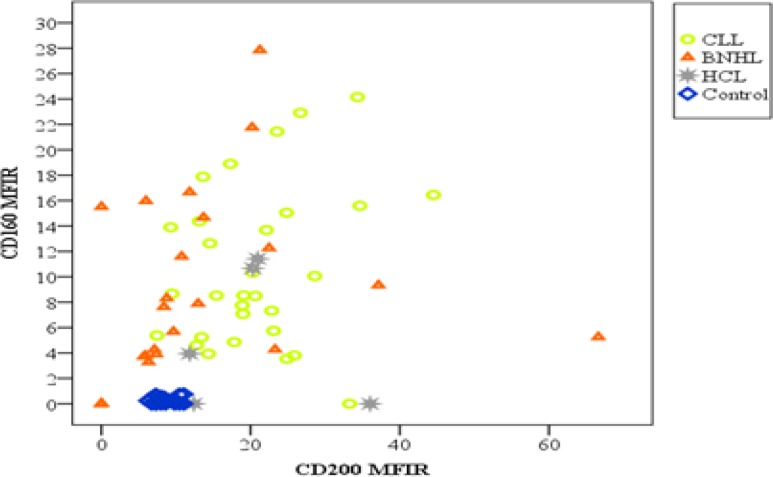
Distribution of patients (CLL and other MBN) and control according to CD 160 and CD200 MFIR.

Regarding matutes scoring, 24 (80%) of CLL patients were 4-5 score and 25 (83.3%) of MBN were 0-2 score. The remaining 11 cases were scored ^3^. All the 6 CLL cases which scored 3 were positively co-expressing CDs160/200 by % and MFIR. On the other hand, only 1 from the 5 B-NHL cases that were score 3 was positive for CD160% and MFIR, while the other 4 cases were double negative by % and MFIR. 

A correlation study was done between CDs160/CD200 expression and different laboratory and clinical parameters, including hemoglobin level, platelet count, total leucocytic count, the absolute lymphocytic count, Rai staging system and matutes score and no statistically significant correlation was found between any of these parameters (p>0.05).

On studying the correlation between CD160 and CD200 as regards both % expression and MFIR, no significant correlation was detected.

Regarding CD160, we found that 88.3 %( 53/60) of our cases were either double positive in percentage expression and ratio or double negative in percentage expression and ratio (results are consistent) and only 11.7 % (7/60) were positive by ratio and negative by percentage. No cases were positive by percent and had negative MFIR. 

On studying CD200, we found that only 41.5%(25/60) of our cases showed consistent positivity or negativity to both percentage expression and MFIR, while 58.5%(35/60) of the cases showed discordant results were 50% of the cases were positive by percentage and negative by ratio and 8.5% were positive by ratio and negative by percentage. 

From the above findings and the fact that the MFIR was more consistent with the % expression in CD160 only rather than the CD200 and depending on our findings regarding the ROC curves sensitivity and specificity, we had to rely on the % expression in detecting the positivity rather than the MFIR (Table 2)**.**

## Discussion

Our study aimed to evaluate CDs160/CD200 expression in CLPD that is presented in leukemic phase which can be misdiagnosed as CLL when atypical clinical, morphological, histochemical, immunophenotypic and/or molecular presentation are affected.

In our study, 90% of patients with CLL had positive CD160 expression on B-cell clones by percentage versus 60% B-NHL and 60% HCL, while by MFIR 96.7% of patients with CLL had positive CD160 B-cell clones versus 80% B-NHL and 60% HCL. 

In agreement with our study, Farren TW et al.^21^ found that CD160 expression was detected in 98% of CLL cases, 100% of HCL, and 16% of other B-LPD cases. They reported that the restriction of CD160 expression to malignant B cells indicates that it is a tumor-specific antigen and an attractive target for the assessment of minimal residual disease in CD160+ B-LPDs.

Lesesve JF et al.^16^ demonstrated that all healthy subjects had MFIR close to one demonstrating that CD160 was not expressed on normal B lymphocytes. In the CLL group, 60 %( 41of 69) of patients had CD160 positive expression, showing statistical significance as compared to the controls (P<0.001). 2(5%) of 42 patients were affected by other MBN expressed CD160 with statistically significant difference when compared to the CLL group (P = 0.0016). 1 of 4 patients suffering from HCL expressed CD160.

The study by Liu FT et al. ^14^ reported that 53 of 54 patients with CLL had CD160 positive expression using the cutoff of more than 20%. 

In contrast to our results, which reported that CD160 expression in MBN was not correlated with any of the clinical data and laboratory data, Zhang ZH et al.^23^ concluded that CD160 expression level in CLL was associated with Binet staging and WBCs count.

In our study, 100% of patients with CLL had positive CD200 expression on B-cell by percentage versus 56% B-NHL and 100% HCL, while 50% of patients with CLL had CD160 B-cell by MFI ratio versus 24% B-NHL and 60% HCL. 

In agreement to our results, Lesesve JF et al.^16^ found that CD200 was not expressed on normal B lymphocytes. In the CLL group, 83% (57 of 69) of patients had CD200 positive expression, showing high statistical significance as compared to the controls (P< 0.001), while 10% (4 of 42) of patients with other MBN showed CD200 expression, and had significant difference when compared to CLL (P<0.0001). 50% (2 of 4) of patients with HCL were positive for CD200.

Spacek M et al. and Gorczynski RM^24^^,^^25^ confirmed previous reports that CD200 is consistently expressed in all typical cases of CLL. Furthermore, CD200 was expressed by all immunophenotypically atypical CLL cases.

Consistent with our results, a study conducted by EL-Desoukey NA et al.^20^ Stated that CD200 was expressed in all CLL patients (100%), and showed higher statistical significance when compared to B-NHL (P<0.001). Also, CD200 was expressed in the 2(100%) cases of HCL. They concluded that the high expression of CD200 in CLL and HCL could open the option for targeted immune (anti-CD200) therapy. 

Poongodi R et al.^26^ reported that CD200 expression was seen in 100% of CLL and HCL patients. On the contrary, CD200 was not expressed on other CLPD except 2 cases (1 MCL and 1FL).

Regarding patients with a Matutes score 3, 6 patients with a diagnosis of CLL had an atypical immunophenotype. All these patients co-expressed CD160 and CD200 by percentage and by MFIR. 5 patients with B-NHL presented with a Matutes score 3. Only one case expressed CD160 by percentage and by MFIR, but none of them had both CDs160/ 200 ratios or percentage expression above defined thresholds; this shows the power of using both markers co-expression in discriminating CLL and other CLPD cases with score 3.

In Lesesve JF et al. ^16^, 3 patients with CLL had an atypical immunophenotype. The leukemic cell of these patients expressed CDs160/CD200 with a MFIR >2, ≥20, respectively. Thus, the 3 showed positive co-expression. 15 patients with non-CLL B-cell neoplasms had score 3 and none of them had positive co-expression of CDs160/200.

Interestingly, we found that co-expression of CDs160/CD200 was found in 90% of CLL and in 60% of HCL compared to 40% of other B-NHL patients; moreover, 24% of B-NHL patients showed double negative expression of both markers.

According to the above presented data and other studies in the literature, CD160 and CD200 are sensitive markers for CLL. This finding recommends using CD160 in combination with CD200 to increase their sensitivity as an additional diagnostic tool to differentiate between CLL and B-NHL. 

Moreover, they can be used to confirm the diagnosis of HCL that may lack any of CD11c, CD25 or CD103.

## CONCLUSION

 Flow cytometric expression of CD160 in combination with CD200 can be used as additional diagnostic markers in the routine panel to differentiate between B-CLL and B-NHL.
